# The use of tafasitamab in diffuse large B-cell lymphoma

**DOI:** 10.1177/20406207211027458

**Published:** 2021-07-06

**Authors:** Johannes Düll, Max Topp, Gilles Salles

**Affiliations:** Medizinische Klinik und Poliklinik II, Universitätsklinik Würzburg, Josef-Schneider-Straße 2, Würzburg, 97080, Germany; Medizinische Klinik und Poliklinik II, Universitätsklinik Würzburg, Würzburg, Germany; Memorial Sloan Kettering Cancer Center, New York, NY, USA

**Keywords:** anti-CD19, clinical data, diffuse large B-cell lymphoma, DLBCL, lenalidomide, mode of action, monoclonal antibody, real-world evidence, tafasitamab

## Abstract

Patients who relapse or are refractory after first-line therapy for diffuse large B-cell lymphoma (DLBCL) frequently have poor prognoses, especially when they are not candidates for autologous stem cell transplant (ASCT). Tafasitamab is a humanized monoclonal anti-CD19 antibody that has recently been approved by the FDA in combination with lenalidomide for the treatment of relapsed/refractory (R/R) DLBCL in patients who are not eligible for ASCT. Tafasitamab has an Fc region which has been modified to have an increased affinity for Fcγ receptors, to potentiate antibody-dependent cellular cytotoxicity and antibody-dependent cell-mediated phagocytosis. Here, we review the development, mode of action and clinical data for tafasitamab in combination with lenalidomide in R/R DLBCL, and discuss the various ways in which this novel antibody could be utilized in the treatment sequence to improve clinical outcomes for patients with DLBCL.

## Introduction

As the most common aggressive non-Hodgkin’s lymphoma (NHL) subtype, diffuse large B-cell lymphoma (DLBCL) accounts for approximately 40% of NHL cases, with a median age at diagnosis of 66 years, and initial treatment is of curative intent.^1,2^ First-line treatment predominantly consists of six cycles of R-CHOP (rituximab, cyclophosphamide, doxorubicin, vincristine and prednisone) chemotherapy, which is ultimately successful in 50–70% of patients.^3^ However, the outlook for patients who are refractory to or relapse following R-CHOP is not promising; although younger and fit patients can receive salvage chemotherapy and autologous stem cell transplant (ASCT), its benefit is limited.^4^ Elderly patients are frequently ineligible for high-dose chemotherapy and ASCT due to considerable toxicity, which is an important consideration given the aging patient population and age at diagnosis.^5^ Over the past 5 years, attention has turned from salvage chemotherapy to immunologic approaches to provide additional, effective alternatives for patients with relapsed/refractory (R/R) DLBCL.

CD19 is a transmembrane protein expressed on B-cells from early in their development and is highly conserved throughout their maturation; it is one of the most reliable surface markers of B-cells.^6^ In normal B-cell development, CD19 has a role in modulating B-cell receptor (BCR) and independent developmental signaling thresholds, and influences both antigen-independent and immunoglobulin-induced B-cell activation, *via* protein kinases including Src, Ras, Abl, Btk, adapter molecules and PI3K.^6,7^ In malignant B-cells, CD19 may be an essential contributor to the chronic activation of BCR and CD40 signaling to drive B-cell lymphomagenesis, survival and proliferation.^8^ CD19 engagement is also known to augment Myc levels and amplify Myc functioning in murine lymphoma cells.^9^

CD19 has emerged as a valuable target in DLBCL, being expressed more broadly than CD20 (the target for rituximab) in B-NHL, and is expressed in patients with CD20 downregulation following rituximab exposure.^10^ Several different approaches have been developed to exploit CD19 on B-cells in patients with R/R DLBCL over the past 5 years, including chimeric antigen receptor T-cell therapy (CAR-T), bispecific antibodies which localize T-cells to CD19, antibody-drug conjugates which deliver a cytotoxic payload to CD19-bearing cells and now tafasitamab in combination with lenalidomide.^11–15^

## Tafasitamab development, structure, mechanism of action (MOA) and early clinical data

Initial attempts to exploit CD19 *via* murine anti-human CD19 monoclonal antibodies (mAbs), with or without linked toxins, were met with limited success, partly as a result of CD19 internalization following antibody binding and the development of human anti-murine antibodies during treatment.^10,16^ The second generation of CD19-targeting antibodies utilized computational algorithms and high-throughput screening to design and select antibodies with specific engineered Fc variant regions to enhance immune effector functions, including antibody-dependent cell-mediated cytotoxicity (ADCC).^17^ Immune effector functions are triggered *via* the interaction of CD19-bound mAb Fc with effector cell Fcγ receptors (FcγRs), resulting in immune responses including natural killer (NK) cell activation, cytotoxic attack and the release of inflammatory mediators.^17,18^ Tafasitamab is one such engineered mAbs, which incorporates S239D and I332E mutations^17^ into the Fc region of humanized anti-CD19 immunoglobulin G.^18^ The S239D/I332E combination demonstrated preclinical enhancement of affinity for FcγRIIIa when engineered into mAbs for a variety of targets.^17^ These effects were replicated with a S239D/I332E in a humanized anti-CD19 mAb, which demonstrated highly enhanced ADCC against several lymphoma and leukemia cell lines in addition to increased antibody-dependent cell-mediated phagocytosis (ADCP) and antiproliferative activity in murine xenograft models.^18^ These effects were further investigated in chronic lymphocytic leukemia (CLL) patient cells, revealing the importance of enhanced activation of NK-cells as immune effectors,^19^ as well as superior ADCC against acute lymphoblastic lymphoma (ALL) patient blast cells, compared with alemtuzumab, rituximab and ofatumumab,^20^ again with a significant role for NK-cells.^21^

Tafasitamab monotherapy was initially investigated with encouraging efficacy in a phase I dose-escalation study in patients with R/R CLL.^22^ No maximum tolerated dose was identified, and tafasitamab was well tolerated at the highest (recommended phase II) dose studied (12 mg/kg each week, with an additional dose on day 4 of cycle 1). The most common adverse events (AEs) observed were Grade 1–2 infusion reactions in 67% of patients, with the most common Grade 3–4 hematologic AEs (neutropenia and thrombocytopenia) occurring in ⩽10% of patients; there was no evidence of immunogenicity. The early efficacy signals reported were a partial response (PR) rate of 67%, and a stable disease rate of 33%; the PR rate was 30% by International Workshop on Chronic Lymphocytic Leukemia 2008 criteria (including response by computed tomography).^22^

Clinical activity with tafasitamab monotherapy was also observed in patients with R/R NHL across indolent and aggressive subtypes, including DLBCL, in a phase II study.^23^ Response rates of 20–30% were observed across subtypes, with an objective response rate (ORR) of 25.7% [95% confidence interval (CI) = 12.5–43.3] in 9 out of 35 patients with DLBCL [seven PR and two complete responses (CRs)], with a median duration of response (DoR) of 20.1 months (95% CI = 1.1–not reached).^23^ Interestingly, in this study, an exploratory *post hoc* analysis found that progression-free survival (PFS) was longer for patients with a baseline peripheral NK-cell count above a threshold of 100 cells/μl.^24^

## Lenalidomide MOA and activity in DLBCL

Lenalidomide is a thalidomide derivative immunomodulatory drug with noted anti-tumor activity across a range of hematologic malignancies.^25^ It has long been acknowledged to have various immunologic effects related to enhancing anti-tumor NK- and T-cell activity, altering the balance of pro- and anti-inflammatory cytokines in the tumor microenvironment (TME), inhibition of angiogenesis, and, to a lesser extent, induction of cell cycle arrest and apoptosis.^25^ Lenalidomide has several direct effects on malignant B-cells, including increased cell cycle arrest in the G_0_–G_1_ phase, decreased cellular proliferation and downregulated expression of checkpoint inhibitors (including PD-L1).^26^ In the TME, lenalidomide stimulates the proliferation and activation of NK-cells, enhancing NK-cell-mediated cytotoxicity and ADCC as a result of enhanced FcγR signaling from bound antibodies such as rituximab. In addition, lenalidomide stimulates the activation and proliferation of T-cells (CD8+ and CD4+) and improves immune synapse formation between malignant B-cells, antigen-presenting cells and effector cells, including NK- and T-cells. Lenalidomide also decreases the production of pro-inflammatory cytokines from T-cells (e.g. TNF-α, IL-1, IL-6 and IL-2) and increases the production of anti-inflammatory IL-10, thereby making the TME less supportive of tumor growth, metastasis and chemoresistance.^26^

In a pooled analysis of early trials of lenalidomide monotherapy in heavily pre-treated patients with R/R DLBCL (*N* = 134), therapy was associated with an ORR of 26% (including 9% CRs) and a median DoR of 6.0 months; a separate retrospective analysis of cell-of-origin found that lenalidomide was more effective in non-germinal center B cell DLBCL than germinal center B (GCB) DLBCL.^27^ In terms of safety, the most common Grade ⩾3 AEs generally associated with lenalidomide monotherapy are neutropenia (~30–40%) and thrombocytopenia (~20%), with lower rates of leukopenia (7–14%) and anemia (6–9%).^27^

Combination therapy with lenalidomide in DLBCL has been more successful. Lenalidomide has been utilized in combination with rituximab in R/R DLBCL in phase II studies including 32^28^ and 23 patients^5^ with moderate efficacy, including an ORR of 28% and 35%, respectively, and CR rates of 22% and 30%, respectively. R-lenalidomide may also be feasible as a bridge to stem cell transplant; in the former study, five of nine responders with DLBCL went on to achieve a CR with stem cell transplant.^28^ In untreated DLBCL, despite promising phase II results in unselected DLBCL patients,^29^ a phase III, randomized study assessing R-CHOP with or without lenalidomide failed to show a benefit for R-CHOP plus lenalidomide in patients with activated B-cell DLBCL (assessed using gene expression profiling).^30^ However, lenalidomide maintenance for 24 months in elderly patients who responded to first-line R-CHOP was found to provide significant improvements in PFS compared with placebo [*N* = 650; PFS not reached (NR) *versus* 58.9 months; hazard ratio (HR) = 0.708 (95% CI = 0.537–0.933); *p* = 0.0135], but with no significant change in overall survival (OS).^31^ In aggressive B-cell lymphomas, lenalidomide combined with the anti-CD20 mAb obinutuzumab prompted the reversal of immature NK phenotypes and an increased expression of NK-activating receptors.^32^

## Rationale for combining tafasitamab and lenalidomide in DLBCL

The rationale for combining tafasitamab and lenalidomide is based on the stimulation and proliferation of NK-cells by lenalidomide, coupled with the amplification of NK-cell-mediated ADCC by tafasitamab.^33^ DLBCL is a disease well-suited to this approach, and low circulating NK-cell levels have been associated with short event-free survival following chemotherapy.^34^ Low NK-cell count at diagnosis (<100/μl) did not affect initial response to therapy, but was associated with significantly poorer PFS and OS outcomes following R-CHOP in a subgroup of DLBCL patients with non-GCB type disease (there was no significant difference with baseline NK count in patients with GCB-type disease), in a retrospective analysis of 72 patients receiving first-line treatment [HR for PFS and OS = 6.03 (95% CI = 1.79–13.55; *p* < 0.001) and 3.75 (95% CI = 1.32–7.72; *p* = 0.005), respectively].^35^ More recently, a retrospective analysis including 1287 patients with DLBCL who received CHOP chemotherapy plus either obinutuzumab or rituximab in the GOYA trial found that a baseline peripheral NK-cell count of <100/μl was associated with significantly shorter PFS (HR = 1.36; 95% CI = 1.01–1.83; *p* = 0.04), and that low tumor NK-cell gene expression was associated with shorter PFS (HR = 1.95; 95% CI = 1.22–3.15; *p* < 0.01) in patients who received obinutuzumab plus CHOP.^36^ Additionally, in a study comparing samples from 36 patients with newly-diagnosed DLBCL with 20 healthy controls, NK-cells from the DLBCL patients were found to have reduced expression of FcγIIIR and reduced degranulation activity when challenged with rituximab-coated tumor cells.^37^ As a result, clinical response to rituximab-based therapy could be affected by impaired rituximab-mediated NK-cell cytotoxicity.^37^

Other observations support the importance of NK-cells in the TME from the perspective of treating DLBCL. In single droplet microfluidic analyses of individual interactions between NK-cells and target B-lymphoma cells, increased NK-cell cytotoxicity was observed with decreased proximity and uninterrupted contact time.^38^ The TME is a crucial regulator of tumor development, and NK-cell inhibition and dysfunction are recognized as important mechanisms of tumor cell escape.^39,40^ In a prognostic gene model of immune cell infiltration and prognosis in DLBCL, the largest cellular factor in the TME that contributed to prognosis was the ratio of activated to resting NK-cells. Higher proportions of activated NK-cells were associated with poorer OS outcomes, indicating NK-cell dysfunction in the TME, and point to reprogramming of activated NK-cells as an important contributor to tumor growth and metastasis.^40^

**Figure 1.**
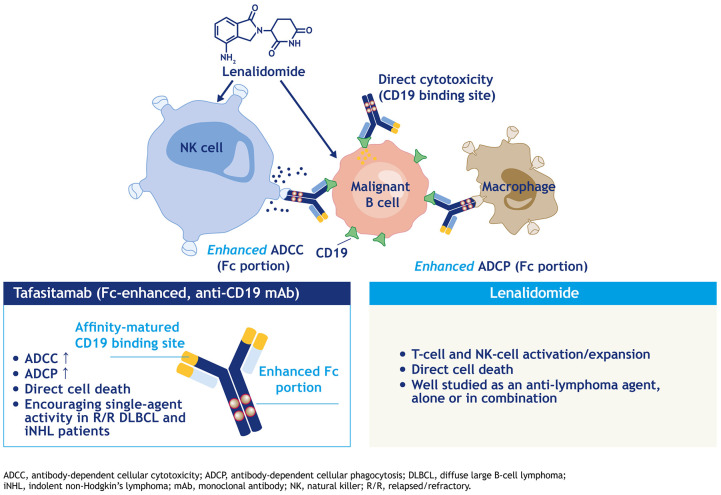
Combination mechanism of action of tafasitamab and lenalidomide.^41^

Through these observational data, the augmentation of NK-cell activity with lenalidomide and the simultaneous exploitation of tafasitamab’s FcγR modifications to further enhance ADCC, ADCP and NK-cell localization to tumor cells *via* CD19 binding (Figure 1) represents a rational and novel combination strategy worth being explored in DLBCL.

## Tafasitamab plus lenalidomide in DLBCL: L-MIND and RE-MIND

The combination strategy for tafasitamab plus lenalidomide is under investigation in the single-arm, open-label phase II L-MIND study (NCT02399085).^33^ Eighty-one patients with R/R DLBCL who had received 1–3 prior regimens, including at least one anti-CD20 regimen, and who were not candidates for high-dose chemotherapy and subsequent ASCT, were enrolled. Patients had a median age of 72 years and a median of 2 prior lines of therapy (range 1–4); all had received R-CHOP (or equivalent chemoimmunotherapy). Nearly half (46%) of patients were aged >70 years, and three-quarters (75%) had stage III/IV disease; 47% of patients had immunohistochemistry-confirmed GCB-type disease. Few patients (11%) had received prior ASCT. Primary refractory disease was present in 19% of cases, with 42.0% and 44.4% of patients refractory to rituximab and last line of therapy, respectively. Although presence of known double- and triple-hit lymphoma was an exclusion criterion for L-MIND, two patients (one double-hit and one triple-hit) were found to have these alterations after enrollment.^33^

**Figure 2.**
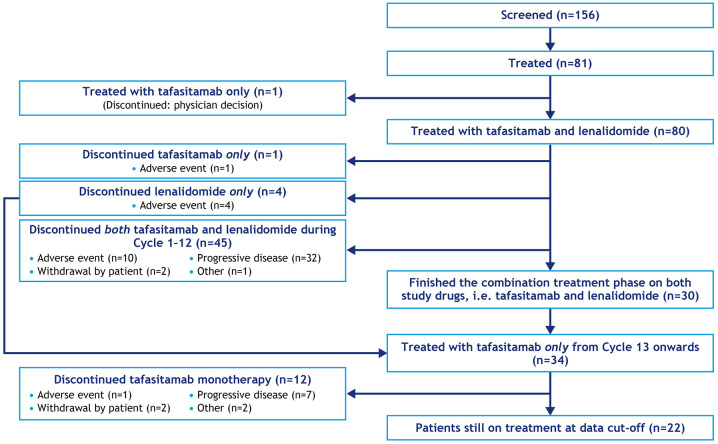
L-MIND schema.^43^

**Figure 3.**
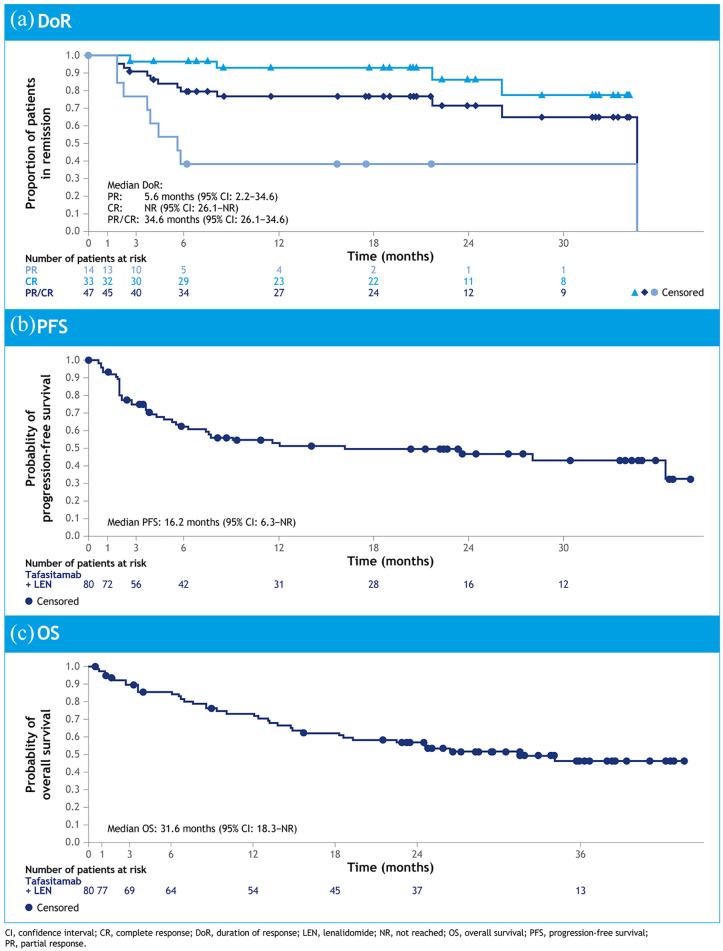
Kaplan–Meier curves for (a) duration of response, (b) PFS and (c) OS.^43^

Eighty patients received combination therapy with tafasitamab 12 mg/kg intravenously once weekly and lenalidomide 25 mg/day orally on days 1–21 for up to 12 28-day cycles, followed by tafasitamab monotherapy (every 2 weeks in patients with stable disease or better) until disease progression (one patient of the 81 enrolled did not receive both agents;^33^ Figure 2). At long-term follow-up (⩾24 months after last patient enrolled),^42^ the ORR was 57.5% (*n* = 46/80), including CR in 40.0% of patients (*n* = 32/80) (Table 1). Median DoR was 34.6 months (95% CI = 26.1–34.6); median OS was 31.6 months (18.3–NR); and median PFS was 12.1 months (6.3–NR). Median DoR was not reached in patients with CR (95% CI = 26.1–NR months), and consistent ORRs were seen in subgroups of patients with primary refractory (53.3) and last-therapy refractory (60.0%) disease [Figure 3(d)].^42^ Responses were also observed in the two patients with double- or triple-hit lymphoma (PR and CR, respectively).^33^

**Table 1.** ORR and CRR in the primary and long-term analyses of L-MIND.

	Tafasitamab plus lenalidomide *N* = 80^b^	
	Primary analysis Data cut-off: 30 November 2018^33^	Follow-up analysis Data cut-off: 30 November 2019^42^
Best objective response, *n* (%)		
CR	34 (43)	32 (40)
PR	14 (18)	14 (18)
ORR – CR + PR; *n* (%) (95% CI)^a^	48 (60) (48–71)	46 (58) (45.9–68.5)
Median DoR – IRC; months (95% CI)	21.7 (21.7–NR)	34.6 (26.1–34.6)
Median PFS – IRC; months (95% CI)	12.1 (5.7–NR)	12.1 (6.3–NR)
Median OS, months (95% CI)	NR (18.3–NR)	31.6 (18.3–NR)

a Using the two-sided 95% Clopper–Pearson exact method based on a binomial distribution.

b One patient received tafasitamab only and was excluded from 81 enrolled patients.

CI, confidence interval; CR, complete response; CRR, complete response rate; DoR, duration of response; IRC, independent review committee; NR, not reached; ORR, objective response rate; OS, overall survival; PFS, progression-free survival; PR, partial response.

**Figure 4.**
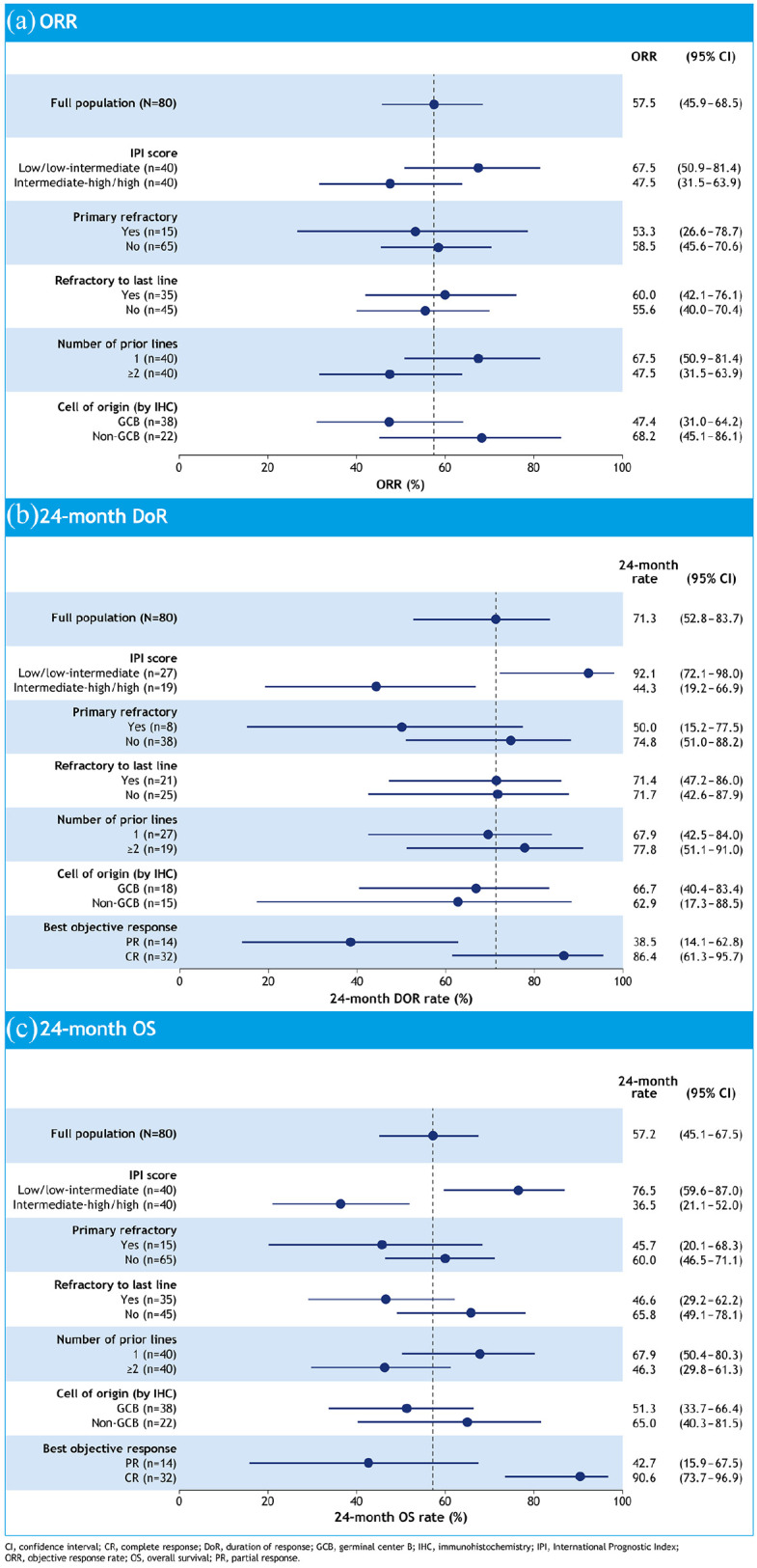
Forest plots for (a) ORR, (b) 24-month duration of response and (c) 24-month OS.^42^

Analysis of time-to-event endpoints [Figure 3(a) to (c)] was encouraging, with the Kaplan–Meier curve for PFS including a potential plateau section after around 12 months [Figure 3(b)], for approximately 40% of patients.^43^ As expected, 24-month OS was shorter in patients with primary refractory disease, although ORR in refractory subgroups was consistent with the rest of the population [Figure 4(a)]. Of the refractory subgroups, primary refractoriness appeared to have a negative impact on both 24-month DoR and 24-month OS, while refractoriness to last therapy had little impact on 24-month DoR [Figure 4(b) and (c)]. Of the other patient subgroups analyzed, the only characteristic to stand out as a consistent marker of poor prognosis was intermediate–high or high-risk International Prognostic Index score at baseline.^42^ Patients who received tafasitamab plus lenalidomide as second-line therapy experienced an improved ORR [67.5% (95% CI = 50.9–81.4) *versus* 47.5% (95% CI = 31.5–63.9)] and 24-month OS [67.9% (95% CI = 50.4–80.3) *versus* 46.3% (95% CI = 29.8–61.3)] compared with those who received the combination as third-or-later-line therapy.^42^

**Figure 5.**
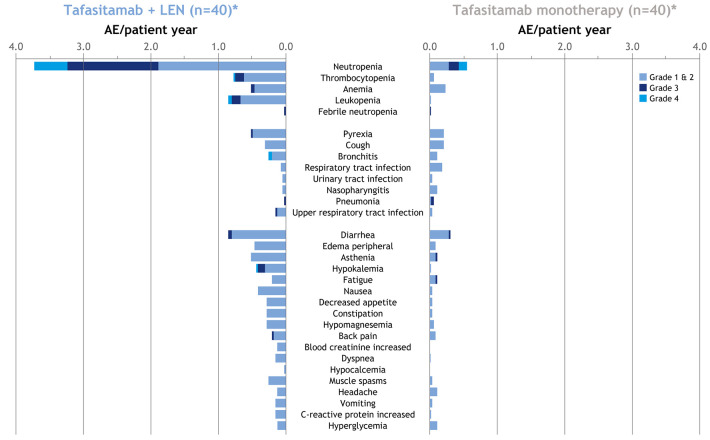
AEs per patient-year during combination and monotherapy phases.^43^ *n = 40 includes 30 patients who completed 12 cycles of tafasitamab plus lenalidomide and continued tafasitamab monotherapy and 10 patients who discontinued lenalidomide but continued tafasitamab monotherapy. AE, adverse event; LEN, lenalidomide.

At long-term follow-up, AEs were consistent with the primary analysis^33^ and with the toxicity profiles of each drug. The primary hematologic AEs were neutropenia (49.4% Grade ⩾3) and thrombocytopenia (17.3% Grade ⩾3), which were both manageable with standard therapy. The incidence of hematologic and non-hematologic AEs declined in the tafasitamab monotherapy phase, following cessation of lenalidomide (Figure 5). Several patients discontinued one or both agents during the combination phase (50 patients; 60%). The majority of discontinuations (32/50; 64%) resulted from disease progression, and 15 patients (18.8%) discontinued one or both drugs due to AEs [the remaining discontinuations were owing to patient withdrawal (*n* = 2) and other (*n* = 1)]. Of the 34 patients who received tafasitamab for >12 months (30 patients who completed 12 months’ lenalidomide and four patients who discontinued lenalidomide early but received tafasitamab for ⩾12 months in total), there was one discontinuation due to AEs (2.9%), seven (20.6%) owing to disease progression, two (5.9%) patient withdrawals and two discontinuations for other reasons (5.9%).^43^

Although data from L-MIND are encouraging, as a single-arm study it cannot delineate the contribution of tafasitamab to the efficacy of combination therapy. Following discussions with the Food and Drug Administration (FDA) to estimate the added efficacy of combination therapy, the observational real-world retrospective cohort RE-MIND (NCT04150328) study of lenalidomide monotherapy was conducted to generate a patient-level matched comparator for the L-MIND study.^44,45^ In this real-world evidence approach, a pool of 140 patients with R/R DLBCL were eligible for matching by receiving lenalidomide monotherapy with a starting dose of 25 mg/day, having at least 6 months’ follow-up data and fulfilling eligibility criteria aligned with L-MIND. The retrospective monotherapy cohort was then balanced for nine prognostically important baseline covariates to represent patients similar to those enrolled in L-MIND, using estimated propensity score-based nearest neighbor 1:1 matching. Cohorts of 76 patients were identified from each study (five patients from L-MIND were not eligible for matching but were included in sensitivity analyses) and outcomes were compared. A significantly higher ORR (primary endpoint) of 67.1% (combination therapy) *versus* 34.2% (lenalidomide monotherapy; *p* < 0.0001) was predicted, together with significant increases in OS and PFS for combination therapy (Table 2).^45^ Efficacy data for the lenalidomide monotherapy cohort in RE-MIND were similar to published prospective trial data with lenalidomide monotherapy,^45–48^ making the RE-MIND cohort a realistic comparator for L-MIND. Although not a substitute for a conventional randomized study, real-word evidence makes an important contribution to drug development and clinical research. The RE-MIND study design included multiple predefined sensitivity analyses to detect and mitigate sources of bias, including repeat analyses of the data with more stringent patient-matching criteria, and additional prognostic factors (e.g. Eastern Cooperative Oncology Group performance status) as balancing covariates, all of which supported the primary analysis.^45^

**Table 2.** Predicted outcomes with tafasitamab plus lenalidomide and lenalidomide monotherapy in the RE-MIND study.^45^.

Predicted outcome	Tafasitamab plus lenalidomide cohort L-MIND regimen; *n* = 76	Lenalidomide monotherapy cohort RE-MIND; *n* = 76	Comparison (95% CI); *p*-value
ORR, % (95% CI)	67.1 (55.4–77.5)	34.2 (23.7–46.0)	OR = 3.9 (1.9–8.1); *p* < 0.0001
CRR, % (95% CI)	39.5 (28.4–51.4)	13.2 (6.5–22.9)	–
Median OS, months^a^	NR	9.4	HR = 0.499 (0.317–0.785); *p* = 0.0026
Median PFS, months^b^	12.1	4.0	HR = 0.463 (0.307–0.698); *p* = 0.0002

a Median follow-up for OS = 21.5 months (tafasitamab + lenalidomide) and 20.9 months (lenalidomide).

b Median follow-up for PFS = 19.7 months (tafasitamab + lenalidomide) and 12.6 months (lenalidomide).

CI, confidence interval; CRR, complete response rate; HR, hazard ratio; NR, not reached; OR, odds ratio; ORR, overall response rate; OS, overall survival; PFS, progression-free survival.

Therefore, tafasitamab plus lenalidomide is an effective option in patients with R/R DLBCL who are not eligible for ASCT, with tafasitamab making a clinically significant contribution to the efficacy of the combination. Tafasitamab was approved by the FDA in combination with lenalidomide for the treatment of patients with R/R DLBCL not eligible for ASCT on the strength of overall response data from L-MIND.^49^ This indication is approved under accelerated approval based on overall response rate, and continued approval for this indication may be contingent upon verification and description of clinical benefit in a confirmatory trial(s).

Beyond evaluation with lenalidomide monotherapy, comparisons with more routinely used treatment regimens, such as rituximab-lenalidomide, would provide greater context for a role for tafasitamab plus lenalidomide in the treatment of patients with R/R DLBCL; however, large randomized data sets comparing standard systemic treatment approaches in R/R DLBCL are lacking. To further assess the clinical utility of tafasitamab plus lenalidomide within the broader treatment landscape for R/R DLBCL, another observational retrospective cohort study, RE-MIND2 (NCT04697160), will compare the efficacy outcomes of the L-MIND cohort with those of matched patient populations treated with systemic NCCN/ESMO guideline listed regimens administered in routine clinical care.

## Open questions for CD19-directed therapy in DLBCL: treatment sequencing and compatibility with CAR-T

The availability of multiple therapies that target the CD19 receptor, including tafasitamab, CAR-T products and the antibody–drug conjugate loncastuximab tesirine, raises the important question of treatment sequencing. Given that exposure to rituximab has long been associated with B-cell CD20 modulation and consequent resistance to subsequent CD20-based therapy,^50,51^ it is possible that CD19-targeted therapies may be affected in a similar manner. Reduced CD19 expression following CAR-T therapy has been observed as a tumor escape mechanism resulting in relapse, likely as a result of genetic post-translational mechanisms rather than modulated CD19 expression, and may preclude further CD19-targeted therapy.^52^ In patients with B-ALL, relapse with loss of CD19 antigen following CD19-targeted CAR-T therapy has been linked to a range of genetic mutations in the exons coding for the transmembrane portion of CD19, which would necessitate the use of alternative targets for further therapy.^53^

Data for other CD19-targeted therapies have been more promising: in a study of 14 patients with DLBCL who received loncastuximab tesirine, CD19 positivity by immunohistochemistry was not affected by treatment, with CD19-directed CAR-T therapy possible after a median 120 days from antibody-drug conjugate failure.^14^ Extensive loss of CD19 expression has not been associated with tafasitamab in CLL cells.^18^ Limited clinical data support the maintenance of CD19 expression following tafasitamab treatment, with one report of a CR to CAR-T therapy after participation in the L-MIND study.^33^

Sequencing of therapies in R/R DLBCL is a developing field now that several alternatives exist outside of traditional chemotherapy. Tafasitamab–lenalidomide and antibody–drug conjugates are regimens that have the advantage of being immediately available ‘off the shelf’ without the manufacturing and pre-conditioning process necessary for CAR-T, and could, therefore, be an option for patients with highly active disease for whom immediate treatment is vital. The continuous tafasitamab–lenalidomide regimen is a departure from more usual fixed-duration regimens in DLBCL, but continuous therapy is not unusual in other fields of oncology and it is likely that long-term efficacy and tolerability will ultimately determine their acceptability to patients.

Tafasitamab–lenalidomide could have several places in the treatment paradigm for R/R DLBCL: for the treatment of transplant-ineligible patients, as a bridge to ASCT, and, which may also be worth exploring, as a bridge to CAR-T. While tafasitamab has been shown to not impair CAR-T activity *in vitro*,^54^ the duration of CD19-masking by tafasitamab *in vivo* should be investigated as it may hinder an immediate switch to a different CD19-targeted therapy immediately post-tafasitamab administration. Further research into the detection of CD19 masking is necessary; staining by fluorescence-activated cell sorting may not be capable of detecting tafasitamab-masked CD19 and immunohistochemistry methods may return false positives due to the detection of the intracellular CD19 domain. New staining methods may, therefore, be required to conclusively determine the duration of tafasitamab occupancy of cell surface CD19.

## Future potential for tafasitamab in DLBCL

R-CHOP has been the standard first-line treatment for DLBCL for over two decades, despite several attempts to improve on it with a variety of approaches.^55^ The FIRST-MIND study incorporates either tafasitamab alone or tafasitamab–lenalidomide alongside R-CHOP as a first-line therapy for DLBCL. Preliminary safety data published at ASH 2020 support the feasibility of this regimen in terms of toxicity, with an expected increased incidence of neutropenia and thrombocytopenia in patients who received lenalidomide.^56^ Efficacy data are imminent.

Other combinations with tafasitamab are also a logical approach: the B-MIND study is a randomized, phase II/III study of rituximab in combination with either tafasitamab or bendamustine in patients with R/R DLBCL who are ineligible for ASCT, and is currently recruiting (NCT02763319^57^). Future combinations of tafasitamab–lenalidomide with bispecific anti-CD20 antibodies, or antibody-drug conjugates, would also be logical steps to enhance responses to simultaneously target CD19 and CD20, and may help prevent disease relapse.

In conclusion, tafasitamab plus lenalidomide is an effective option for patients with R/R DLBCL who are not eligible for ASCT, and tafasitamab itself is a promising combination partner for other therapies. This is an exciting time in the field of DLBCL; while the optimum sequence for CD19-targeting therapies following R-CHOP has yet to be determined, the last 5 years have at least provided several new avenues to explore, including further potential opportunities to improve on R-CHOP as first-line therapy.
